# Omentoplasty and Thoracoplasty for treating postpneumonectomy bronchopleural fistula in a patient previously submitted to aortic prosthesis implantation

**DOI:** 10.1186/1749-8090-4-38

**Published:** 2009-07-24

**Authors:** Mario Nosotti, Ugo Cioffi, Matilde De Simone, Paolo Mendogni, Alessandro Palleschi, Lorenzo Rosso, Michele M Ciulla, Luigi Santambrogio

**Affiliations:** 1 Department of Surgery-Thoracic and Transplant Unit, Fondazione IRCCS Ospedale Maggiore Policlinico, Milano; Università degli Studi di Milano, Milan, Italy; 2 Department of Surgery, Fondazione IRCCS Ospedale Maggiore Policlinico, Milano; Università degli Studi di Milano, Milan, Italy; 3 Department of Respiratory and Cardiovascular Disease, Fondazione IRCCS Ospedale Maggiore Policlinico, Milano; Università degli Studi di Milano, Milan, Italy

## Abstract

Bronchopleural fistula following pneumonectomy is a serious and frightening complication in chest surgery with a high mortality rate. The possibility of curing this complication using a conservative treatment is extremely poor. Below we describe a case of a patient affected by left pleural empyema due to a postpneumonectomy bronchopleural fistula. The patient had previously undergone an aortic prosthesis implantation. He was successfully treated using omental pedicle in order to cover the bronchial stump, to fill the pleural space and to protect the aortic prosthesis. He also underwent thoracoplasty to collapse the residual pleural space. The postoperative course was uneventful. During the follow-up, after thirty months, the patient was asymptomatic, and no recurrence of the fistula was present.

## Background

Bronchopleural fistula (BPF) is a serious and frightening complication of pulmonary surgery with a high mortality rate [1]. Different methods have been used to close the fistula; from conservative treatment such as bronchial gluing or stent placement [1], to surgical management [2,3].

We report a case of postpneumonectomy BPF successfully treated using omental pedicle and thoracoplasty in a patient with previously aortic prosthesis implantation.

## Case presentation

**Figure 1:**
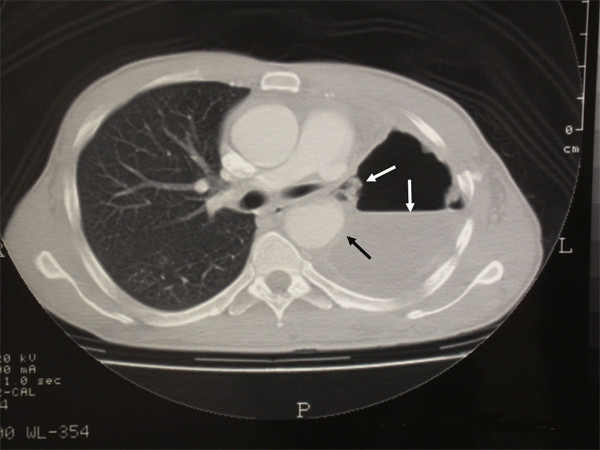
**Axial CT scan (window setting) shows air-liquid level and bronchopleural fistula (white arrows)**. The aortic prosthesis is covered by purulent pleural fluid (black arrow).

In August 2006, a 39-year old man was referred to our department for pleural empyema resulting from a large left main bronchus fistula two months after pneumonectomy. Twenty years before the patient had undergone to aortoplasty with a Dacron patch reconstruction for isthmic aortic stenosis, followed by two thoracotomies for a hemothorax. In July 2006, the patient underwent an aneurysmectomy and prosthesis aortic implantation using a Gelweave™ vascular prosthesis (Terumo Vascutek, Renfrewshire, Scotland, UK) for isthmic aortic aneurysm at the Department of vascular surgery of another hospital. During the operation a left pneumonectomy was undertaken for uncontrolled bleeding drug induced. In the early postoperative period the patient was submitted to two additional re-thoracotomies for serious recurrent left hemothorax. A few days later, the patient presented with fever, chills, malaise, leucocytosis, and purulent pleural fluid from the chest tube due to empyema secondary to the bronchopleural fistula. The patient was transferred to our department. A flexible bronchoscopy revealed the palsy of the left vocal cord due to recurrent laryngeal nerve injury, and a large dehiscence of the left main bronchial stump in the medial portion. A chest CT scan revealed a left empyema and the hyperinflation of the right lung (fig. 1). Firstly, we tried to treat the fistula conservatively using endobronchial apposition of biological glue, and daily pleural antibiotic irrigation until the microbiological assays on pleural fluid became negative. As the patient's general condition had improved, we decided to carry out a surgical treatment. The greater omentum was mobilized from the greater curve of the stomach, through a median laparotomy supported by the left gastroduodenal artery. Subsequently, a left posterolateral thoracotomy was performed. The bronchial stump showed almost complete dehiscence. After the removal of infected and necrotic tissue using sharp debridement and pulsed lavage, the pleural space was filled with antibiotic solution, and the well vascularized pedicle of the greater omentum was transposed into the left hemithorax through the central tendon of the diaphragm. The omental flap was fixed onto the bronchial stump using interrupted sutures and biological glue. Subsequently, we performed a thoracoplasty by resection from the 3^rd ^to the 8^th ^rib. The collapse of the chest wall, including the parietal pleura and intercostal muscles, led to a complete and well-made obliteration of the residual pleural space. A chest tube and a subcutaneous drainage were placed and the thoracic incision was sutured. Finally, an abdominal aspirative drainage was inserted and the laparotomy was closed.

**Figure 2:**
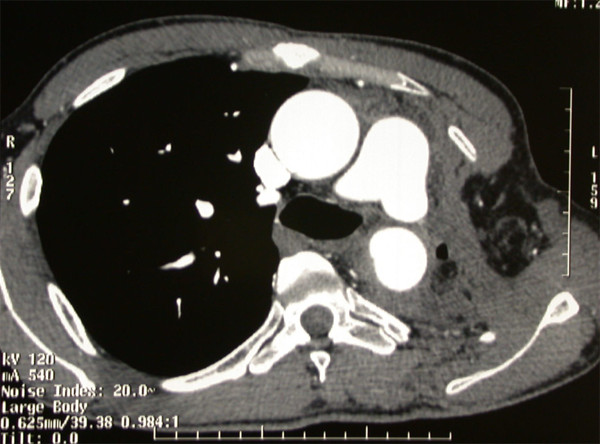
**Axial contrast enhanced CT shows the remodelling osteomuscular wall of the thoracic cage with a collapse of left pleural space**.

The postoperative course was uneventful and the patient was discharged in general good condition 21 days after surgery. A flexible bronchoscopy revealed no recurrence of bronchopleural fistula at 6 and 12 months. A CT, carried out at the same time, showed a complete obliteration of the residual pleural space (fig. 2). After thirty months follow-up no recurrence of the fistula was present.

## Conclusion

Drainage of the infected pleural space, antibiotics to treat infection, and accurate clearance of secretions from the remaining lung should be the initial treatment modality in Stage 1 disease [4,5]. Once the infection is under control, several surgical techniques can be considered in order to cure BPF ranging from omentoplasty, pedicled pericardial fat or pleural flap, myoplasty, thoracoplasty [5]. In our patient we considered the omentoplasty to cover the bronchial stump and to protect the aortic prosthesis, and thoracoplasty to collapse the left pleural space and to control the underlying inflammatory process.

In complex subset we believe that omentoplasty is a reliable approach when attempting to close bronchopleural fistula as also reported by other authors [2,3], since the omentum has the ability to function in the established infected area demonstrated by its natural role in the abdomen [4]. To our knowledge, the case reported is the first in English literature because of the presence of a non-covered aortic prosthesis in an infected pleural cavity, with a very high risk of infection and rupture of the prosthesis.

## Consent

Written informed consent was obtained from the patient for publication of this case report and any accompanying images. A copy of the written consent is available for review by the Editor-in-Chief of this journal.

## Competing interests

The authors declare that they have no competing interests.

## Authors' contributions

All authors read and approved the final manuscript.
